# PBRM1 mutation as a predictive biomarker for immunotherapy in multiple cancers

**DOI:** 10.3389/fgene.2022.1066347

**Published:** 2023-01-09

**Authors:** Jiali Dai, Yanan Cui, Xiao Liang, Jiali Xu, Jun Li, Yu Chen, Erbao Zhang, Renhua Guo

**Affiliations:** ^1^ Department of Oncology, First Affiliated Hospital of Nanjing Medical University, Nanjing, Jiangsu, China; ^2^ Department of Epidemiology, Center for Global Health, School of Public Health, Nanjing Medical University, Nanjing, China; ^3^ Jiangsu Key Lab of Cancer Biomarkers, Prevention and Treatment, Collaborative Innovation Center for Cancer Personalized Medicine, Nanjing Medical University, Nanjing, China

**Keywords:** PBRM1, immune checkpoint inhibitor, biomarker, pan-cancer, immune microenvironment

## Abstract

**Background:** There has been evidence that Polybromo-1 (PBRM1) mutation was closely associated with immunotherapy response in clear cell renal cell carcinoma (ccRCC). However, it remains incompletely unclear whether PBRM1 mutations correlate with ICI response in pan-cancer.

**Methods:** The clinical data and whole exome sequencing (WES) data were collected from seven published immunotherapy studies to evaluate the association between PBRM1 mutation and ICIs efficacy in the discovery cohort. In order to provide further insight into the relationship between PBRM1 and immunity, we analyzed a relatively large sample as a validation cohort. Moreover, we also collected the clinical data and mutation information of 134 non-small cell lung cancer (NSCLC) patients from the First Affiliated Hospital of Nanjing Medical University to verify the findings. Gene set enrichment analysis (GSEA) was used to evaluate the relationship between PBRM1 and immune-related pathway.

**Results:** Our results found that PBRM1 mutation were associated with immune response in the discovery cohort (Progression free survival [PFS]: hazard ratio (HR) = .51, 95% CI: .28–.95, *p* = .030; objective response rate [ORR]: 47.92% vs. 28.21%, *p* = .0044; disease control rate [DCR]: 72.92% vs. 47.53%, *p* = .0008). In the validation cohort, the patients with PBRM1 mutation had a longer overall survival (OS) (hazard ratio = .69, 95% CI: .53–.91, *p* = .006). In our non-small cell lung cancer cohort, PFS, objective response rate and disease control rate had obvious superiority in the patients with PBRM1 mutation than those without PBRM1 mutation (PFS: HR = .268, 95% CI: 084–.854, *p* = .04, ORR: 55.56% vs. 20.00%, *p* = .027, DCR: 100% vs. 75.20%). Using the Gene set enrichment analysis (GSEA) in TCGA cohorts, PBRM1 mutation was closely related to immune efficacy and immune microenvironment, including killer cell mediated immunity regulation, cell cytokine production, CD8^+^ T-cell activation and MHC protein binding process.

**Conclusion:** There is a strong correlation between PBRM1 mutation and prognosis and immune response. Based on the findings, PBRM1 mutation may be a promising immunotherapeutic signature that could guide clinical management and personalized immunotherapy.

## Introduction

Immune checkpoint inhibitors (ICIs), including programmed cell death protein (ligand) 1 [PD-(L)1] and cytotoxic T-lymphocyte antigen-4 (CTLA-4), have gained unprecedented efficacy in multiple cancers (Gandhi et al., 2018; Gutzmer et al., 2020; Marabelle et al., 2020). Nevertheless, only a limited subset of patients have long-lasting clinical benefit of immunotherapy (Hegde and Chen, 2020). Although some predictive indicators such as PD-L1 expression (Gibney et al., 2016), tumor mutation burden (TMB) (Gonzalez-Cao et al., 2018), copy number alteration (CNA) (Liu et al., 2019) and high microsatellite instability (MSI-H) (Zhao et al., 2019) were discovered associated with ICIs response, single biomarker may have limited prediction power. Therefore, the finding of novel therapeutic markers for immunotherapy is urgently required.

Polybromo-1 (PBRM1) is located on chromosome 3p21, which can encode BRG1-associated factor 180 (BAF180), a component of the polybromo-associated BAF (PBAF) chromatin-remodeling complex (Li et al., 2020; Zhou et al., 2020; Feng et al., 2022). PBRM1 is involved in cell differentiation, proliferation and DNA repair in multiple cancers, including clear cell renal cell carcinoma (ccRCC) (Aili et al., 2021; Chabanon et al., 2021), non-small cell lung cancer (NSCLC) (Zhou et al., 2020) and bladder cancer (BLCA) (Lu and Allis, 2017). Although previous studies have reported the possible association of PBRM1 with immune response (Zhou et al., 2020; Aili et al., 2021; Yang et al., 2021; Saiga et al., 2022), the specific role of PBRM1 in the pan-cancer with immunotherapy remains not thoroughly confirmed.

Based on these previous findings, we comprehensively investigated the role of PBRM1 for immunotherapy in multiple cancers. In our study, we observed that PBRM1 mutations are associated with survival prognosis in patients treated with ICIs. Alternatively, we found that PBRM1 mutations showed strong correlation with TMB, immune response and tumor immune microenvironment. These results implied the potential use of PBRM1 mutation as a newly prognostic indicator and therapeutic target for the patients with immunotherapy in multiple cancers.

## Materials and methods

### Clinical cohorts and TCGA cohort

**FIGURE 1:**
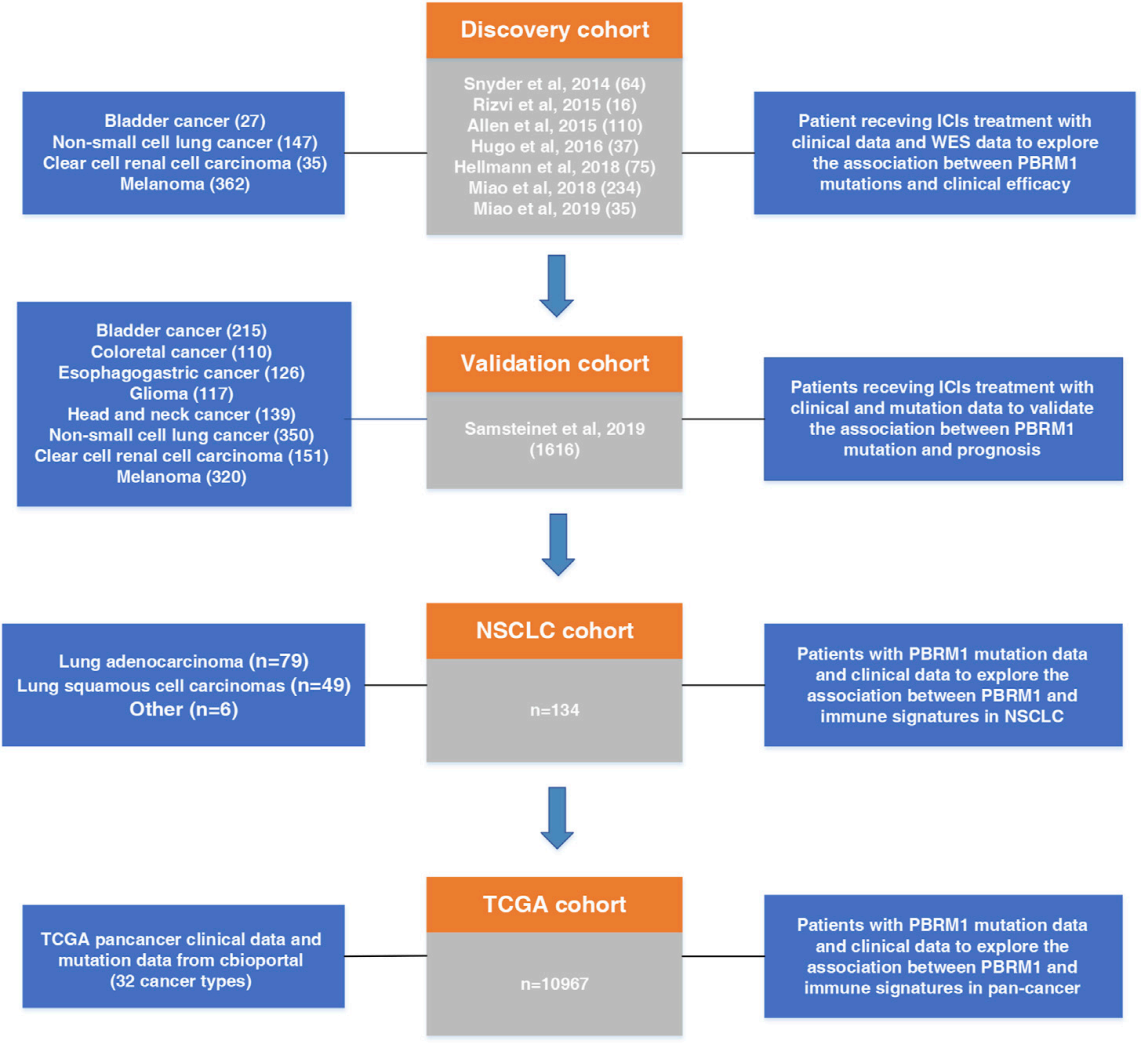
Flowchart of the study design.

As shown in Figure 1, the flow diagram of this study was depicted. To evaluate the predictive functions of PBRM1 in ICI-treated patients, the clinical data and whole exome sequencing (WES) data were collected from seven published immunotherapy studies on cBioPortal (https://www.cbioportal.org) (Snyder et al., 2014; Rizvi et al., 2015; Van Allen et al., 2015; Hugo et al., 2016; Hellmann et al., 2018; Miao et al., 2018a; Miao et al., 2018b). A total of 571 patients from four cancer types were included in the research as the discovery cohort, including BLCA, NSCLC, ccRCC, melanoma (SKCM).

To validate the predictive power of PBRM1 mutations to immunotherapy, a pan-cancer cohort containing survival data and mutational data from cBioPortal was included in our study as the validation cohort (Samstein et al., 2019). Samples from this cohort were collected using the MSK-IMPACT panel. After screening, 1,616 patients from eight cancer types were enrolled in the validation cohort, including BLCA, colorectal cancer (CRC), esophagogastric cancer (ESCA), glioma, head and neck cancer (HNSC), NSCLC, ccRCC, and SKCM.

To investigate the effect of PBRM1 mutation on prognosis, somatic mutation data and genome data in TCGA cohort were retrieved from cBioPortal. RNA-seq data were retrieved from the UCSC Xena data portal.

A total of 134 NSCLC patients who received at least two cycles of immunotherapy from the First Affiliated Hospital of Nanjing Medical University enrolled in NSCLC cohort, including lung adenocarcinoma (LUAD) (79), lung squamous cell carcinomas (LUSC) (49) and other NSCLC (Gonzalez-Cao et al., 2018). All patients had undergone comprehensive genomic profiling of tumor tissue genetic testing before immunotherapy.

### Study assessment

Progression free survival (PFS), overall survival (OS), objective response rate (ORR), and disease control rate (DCR) were the primary clinical outcomes. ORR and DCR was assessed by Response Evaluation Criteria in Solid Tumors (RECIST) version 1.1. In discovery, validation and NSCLC cohorts, PFS and OS were calculated from the date the patient began immunotherapy to the date of progression or death, respectively. In the case of patients without a death or progression, the latest scan was used to censor them. In addition, survival data from TCGA cohort were evaluated from the date of first diagnosis.

### TMB data analysis

TMB was defined as the number of somatic mutations in the exon coding region of the tumor genome that removed germline mutations. All TMB data mainly include non-synonymous mutations. This study defined high and low TMB as the top 20% of high and low TMB for each cancer type in the discovery and validation cohort (Samstein et al., 2019).

### Immune signature and pathway enrichment analysis

To examine the relationship between the gene mutation and the immune microenvironment, the immune cell infiltration level with the different expression groups of PBRM1 were analyzed by the CIBERSORT web portal in TCGA cohort (Newman et al., 2015). Immune-related genes and their functional classification provided by the study from Thorsson et al. (Thorsson et al., 2018). Gene set enrichment analysis (GSEA) was performed to evaluate the relationship between PBRM1 and immune-related pathway.

### Human sample collection and immunohistochemistry

We collected five pairs of tissue samples receiving immunotherapy, and all of patients from the First Affiliated Hospital of Nanjing Medical University. All samples were confirmed as primary NSCLC by pathology, and were sequenced through whole-exome sequencing. Five of the patients were associated with PBRM1 mutations, while the other five were PBRM1 wild-type. Anti-PBRM1 (abconal, Wuhan, China, A0334) and anti-CD8 (Servicebio, Wuhan, China, GB12068) immunohistochemistry (IHC) staining assays were performed on the tissue sections.

### Statistical analysis

All analyses were conducted using customized the R software (version 4.1.3). The Kaplan-Meier method and Cox regression were used to analyze PFS and OS. ORR and DCR was analyzed using Chi-square test or Fisher’s exact test. All comparisons were performed by Wilcoxon rank test. All *p*-values were two-sided and *p*-values of .05 or less were considered statistically significant.

## Results

### Association between PBRM1 mutation and ICIs efficacy in the discovery cohort

**FIGURE 2:**
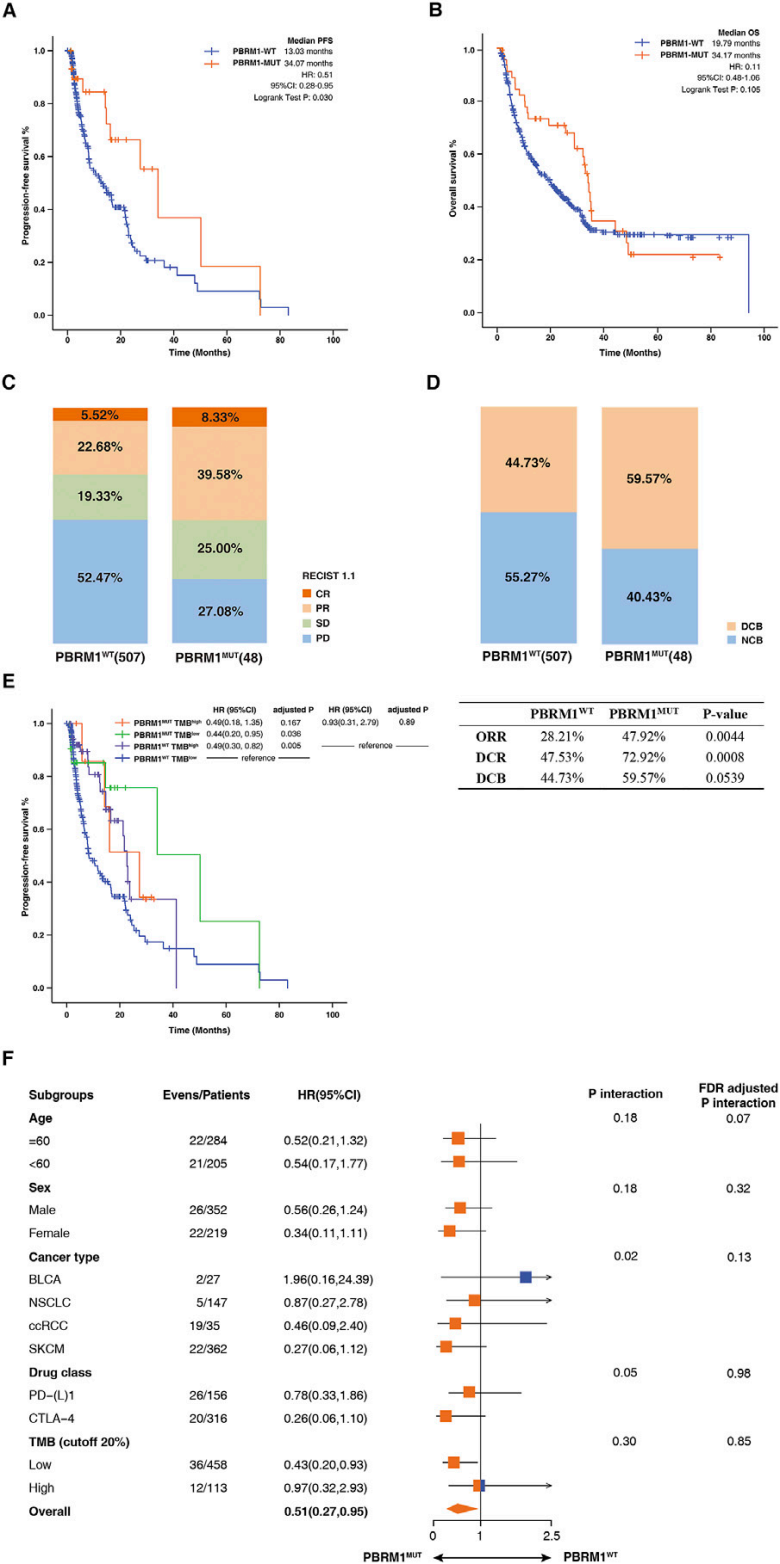
Association between PBRM1mutation and ICIs efficacy in the discovery cohort. **(A)** The Kaplan-Meier survival analysis comparing PFS between PBRM1-MUT and PBRM1-WT patients in the discovery cohort. **(B)** The Kaplan-Meier curves comparing OS between PBRM1-MUT and PBRM1-WT patients in the discovery cohort. **(C)** Histogram depicting proportions of ORR and DCR in PBRM1-MUT and PBRM1-WT patients. **(D)** Histogram depicting proportions of DCB and NCB in PBRM1-MUT and PBRM1-WT patients. **(E)** The Kaplan-Meier curves comparing PFS among PBRM1^MUT^TMB^high^, PBRM1^MUT^TMB^low^, PBRM1^WT^TMB^high^, and PBRM1^WT^TMB^low^ groups in the discovery cohort. **(F)** Subgroup analysis of PFS in the discovery cohort.

We included 571 patients treated with ICIs from seven independent studies as a discovery cohort (Supplementary Table S1). A total of four tumors were included: BLCA (n = 27), NSCLC (n = 147), ccRCC (*n* = 35), SKCM (*n* = 362). 48 patients were carrying PBRM1 mutation (PBRM1-MUT), and 523 patients were PBRM1 wild-type (PBRM1-WT), accounting for over 90% of patients in the discovery cohort. It was found that patients harboring PBRM1-MUT had a longer PFS (median PFS 34.07 months vs. 13.03 months, hazard ratio (HR) = .51, 95% CI: .28–.95, log-rank test *p* = .030, Figure 2A), higher ORR (47.92% vs. 28.21%, *p* = .0044, Figures 2C, D) and higher DCR (72.92% vs. 47.53%, *p* = .0008, Figures 2C, D). The proportion of DCB is 59.57% in PBRM1-MUT patients, which higher than that in the patients with PBRM1-WT. Although *p* values for OS were not statistically different, OS in PBRM1-MUT patients was longer than that in PBRM1-WT group (median OS 34.17 months vs. 19.79 months, HR = .11, 95% CI: .48–1.06, log-rank test *p* = .105, Figure 2B). Relevant studies have shown that high level of TMB in cancer is closely related to tumor malignancy and immunotherapy efficacy. To further evaluate the effect of PBRM1 and TMB on immunotherapy response, patients was divided into four groups: PBRM1^MUT^ TMB^high^, PBRM1^MUT^ TMB^low^, PBRM1^WT^ TMB^high^, and PBRM1^WT^ TMB^low^. As shown in Figure 2E, relatively longer PFS was observed in the PBRM1-MUT group compared to the PBRM1-WT group regardless of TMB level. Notably, PBRM1-MUT could significantly improve the survival of TMB-low patients. Based on these findings, the mutation status of PBRM1 and TMB can jointly affect ICIs efficacy. According to the subgroup analyses, PBRM1-MUT showed an apparent survival advantage over PBRM1-WT, regardless of age, sex, cancer type (except for BLCA), drug class, and TMB level (except for TMB high) (*p* > .05, Figure 2F). However, BLCA patients with PBRM1-WT had longest PFS than PBRM1-MUT patients (Figure 2F). At high TMB, there was no significant difference in PFS between PBRM1-MUT and PBRM1-WT groups (Figure 2F).

### Association between PBRM1 mutation and ICIs efficacy in the validation cohort

**FIGURE 3:**
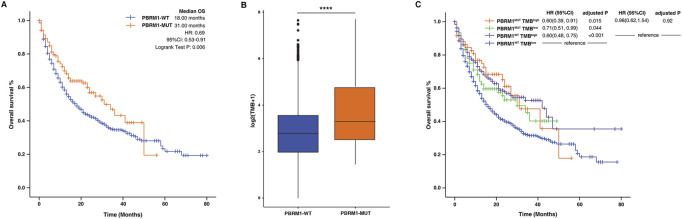
Association between PBRM1mutation and ICIs efficacy in the validation cohort. **(A)** The Kaplan-Meier survival analysis comparing OS between PBRM1-MUT and PBRM1-WT patients in the validation cohort. **(B)** Comparison of the TMB between PBRM1-WT and PBRM1-MUT tumors in validation cohort. **(C)** The Kaplan-Meier curves comparing OS among PBRM1^MUT^TMB^high^, PBRM1^MUT^TMB^low^, PBRM1^WT^TMB^high^, and PBRM1^WT^TMB^low^ groups in the validation cohort. ****p < .0001.

To further confirm the relationship between immunotherapy efficacy and PBRM1 mutation, we analyzed a relatively large sample as the validation cohort. A total of 1,616 patients were included, involving eight types of tumors including BCLC (*n* = 215), colorectal cancer (CRC) (*n* = 110), ESCA (*n* = 126), Glioma (*n* = 117), HNSC (*n* = 139), NSCLC (*n* = 350), ccRCC (*n* = 151), SKCM (*n* = 320). As shown in Table 1, the validation cohort patient characteristics were summarized. The median OS of patients were 18.00 months in PBRM1-WT group and 31.00 months in PBRM1-MUT group (HR = .69, 95% CI: .53–.91, log-rank test *p* = .006; Figure 3A). Moreover, the patients with PBRM1 mutations have higher TMB than the ones without PBRM1 mutations (*p* < .0001, Figure 3B). PBRM1 status and TMB level were used as factors to divide patients into four groups: PBRM1^MUT^ TMB^high^, PBRM1^MUT^ TMB^low^, PBRM1^WT^ TMB^high^, and PBRM1^WT^ TMB^low^. No matter the level of TMB, patients with mutation of PBRM1 had significantly longer PFS than that without PBRM1 mutation. In the existence of TMB high states, the patients with PBRM1 mutations showed a better survival outcome than the ones without PBRM1 mutations. In particular, PBRM1-MUT had a significant positive effect on patients with TMB deficiency (Figure 3C).

**TABLE 1:** Patient characteristics in the validation cohort stratified by PBRM1 status.

Characteristics	NO. (%)	PBRM1 status [no. (%)]^a^	
		PBRM1-MUT	PBRM1-WT
No. of patients	1,616	141	1,475
Median age, years (range)	65 (15–90)	63 (28–90)	63 (15–90)
Age			
≥60	979 (60.58)	91 (64.54)	888 (60.20)
<60	636 (39.36)	50 (35.46)	586 (39.73)
NA^b^	1 (.06)	0 (0)	1 (.07)
Gender			
Male	1,033 (63.92)	99 (70.21)	934 (63.32)
Female	583 (36.08)	42 (29.79)	541 (36.68)
Cancer type			
BLCA	215 (13.30)	7 (4.96)	208 (14.10)
CRC	110 (6.81)	8 (5.67)	102 (6.92)
ESCA	126 (7.80)	6 (4.26)	120 (8.14)
Glioma	117 (7.24)	5 (3.55)	112 (7.59)
HNSC	139 (8.60)	3 (2.13)	136 (9.22)
NSCLC	350 (21.66)	18 (12.77)	332 (22.51)
ccRCC	151 (9.34)	59 (41.84)	92 (6.24)
SKCM	320 (19.80)	30 (21.28)	290 (19.66)
NA^c^	88 (5.45)	5 (3.55)	83 (5.63)
Drug class			
PD-(L)1	1,286 (79.58)	114 (80.85)	1,172 (79.46)
CTLA-4	79 (4.89)	5 (3.55)	74 (5.02)
Combination	251 (15.53)	22 (15.60)	229 (15.53)
TMB (cutoff 20%)			
Low	1,224 (80.10)	77 (54.61)	1,147 (77.76)
High	304 (19.90)	59 (41.84)	245 (16.61)

Abbreviations: NA, not available; BLCA, bladder cancer; CRC, colorectal cancer; ESCA, esophagogastric cancer; HNSC, head and neck cancer; NSCLC, non-small cell lung cancer; ccRCC, clear cell renal cell carcinoma; SKCM, melanoma; PD-(L)1, programmed cell death-1, or programmed deathligand; CTLA-4, cytotoxic T-cell lymphocyte-4.

^a^ Indicated percentage of PBRM1-MUT, or PBRM1-WT, patients in a given category (i.e., specific gender, specific age group).

^b^ Onepatient with age were not reported.

^c^ Eighty-eightpatients with cancer typewere not reported.

### Association between PBRM1 mutation and ICIs efficacy in the NSCLC cohort

**FIGURE 4:**
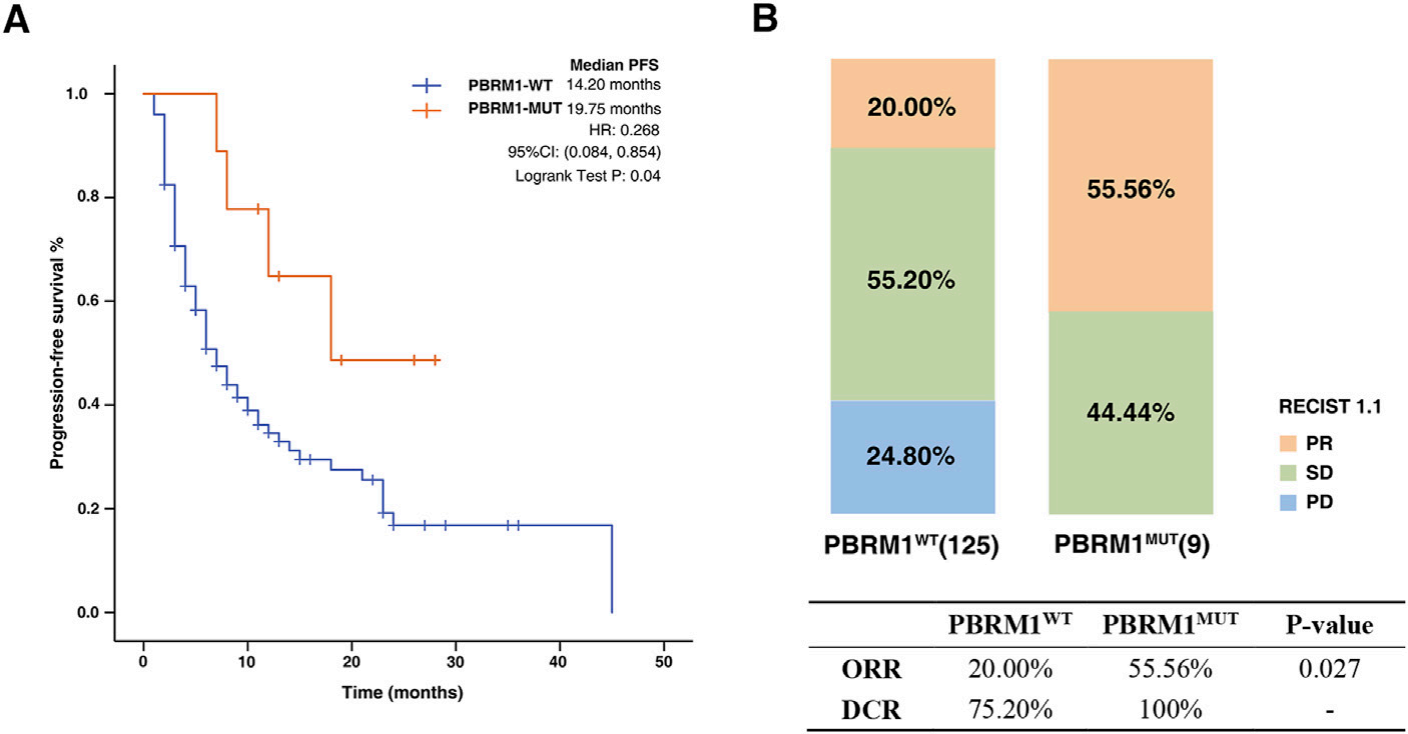
Association between PBRM1 mutation and ICIs efficacy in NSCLC cohort. **(A)** The Kaplan-Meier survival analysis comparing PFS between PBRM1-MUT and PBRM1-WT patients in NSCLC cohort. **(B)** Histogram depicting proportions of ORR and DCR in PBRM1-MUT and PBRM1-WT patients.

There were 134 NSCLC patients from the First Affiliated Hospital of Nanjing Medical University enrolled in our study. Baseline characteristics of the patients included in this investigation are summarized in Table 2. Among these patients, the mutation frequency for PBRM1 was approximately 6.71%. The PFS time was significantly extended in the PBRM1-MUT group compared with that of the PBRM1-MT group (median PFS 19.75 months vs. 14.20 months, HR = .268, 95% CI: 084–.854, log-rank test *p* = .04, Figure 4A). In addition, the ORR and DCR had obvious superiority in the patients with PBRM1 mutation than those without PBRM1 mutation (ORR: 55.56% vs. 20.00%, *p* = .027, DCR: 100% vs. 75.20%, Figure 4B). On the basis of these results, the PBRM1 mutation has an important role in immunotherapy efficacy, which could predict clinical benefit of cancer patients.

**TABLE 2:** Patient characteristics in the NSCLC cohort stratified by PBRM1 status.

Characteristics	NO. (%)	PBRM1 status [no. (%)]^a^	
		PBRM1-MUT	PBRM1-WT
No. of patients	134	9	125
Median age, years (range)	65 (32–81)	68 (52–81)	65 (32–80)
Age			
≥60	96 (71.64)	8 (88.89)	88 (70.40)
<60	38 (28.36)	1 (11.11)	37 (29.60)
Gender			
Male	111 (82.84)	9 (100)	102 (81.60)
Female	23 (17.16)	0 (0)	23 (18.40)
Histology			
LUAD	79 (58.96)	5 (55.56)	74 (59.20)
LUSC	49 (36.57)	2 (22.22)	47 (37.60)
Other	6 (4.48)	2 (22.22)	4 (3.20)
Drug class PD-(L)1	134 (100)	9 (100)	125 (100)
Lines of treatment			
First	71 (52.99)	5 (55.56)	66 (52.80)
Second	27 (20.15)	3 (33.33)	24 (19.20)
Third or subsequent	36 (26.87)	1 (11.11)	35 (28.00)
Stage			
I	4 (2.99)	0 (0)	4 (3.20)
II	4 (2.99)	0 (0)	4 (3.20)
III	31 (23.13)	4 (44.44)	27 (21.60)
IV	95 (70.90)	5 (55.56)	90 (72.00)
Smoking history			
Yes	63 (47.01)	7 (77.78)	56 (44.80)
NO	71 (52.99)	2 (22.22)	69 (55.20)
Best overall response			
PR	30 (22.39)	5 (55.56)	25 (20.00)
SD	73 (54.48)	4 (44.44)	69 (55.20)
PD	31 (23.13)	0 (0)	31 (24.80)

Abbreviations: LUAD: lung adenocarcinoma, LUSC: Lung squamous cell carcinomas, PR: partial response, SD: stable disease, PD: progressive disease, PD-(L)1: programmed cell death-1 or programmed deathligand.

^a^ Indicated percentage of PBRM1-MUT or PBRM1-WT patients in a given category (i.e., specific gender, specific age group).

### Potential mechanisms associated with PBRM1 mutations in predicting the immune response in TCGA cohort

**FIGURE 5:**
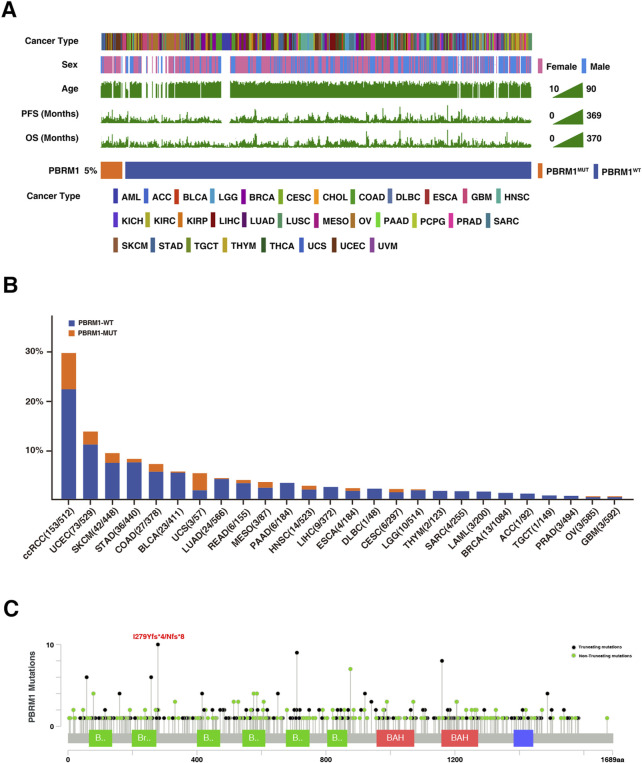
Mutational landscape of PBRM1 in TCGA cohort. **(A)** Association of PBRM1 status and clinical characteristics in TCGA cohort. The cancer type, sex, age, TMB, PFS, and OS were annotated. Samples were sorted by PBRM1 status, while PBRM1-MUT and PBRM1-WT samples were separated by a gap. **(B)** The proportion of PBRM1-MUT tumors identified in each cancer type with at least one mutation case. **(C)** Lollipop plot showing the loci distribution of mutations across the PBRM1 altered patient cohorts from the TCGA database. “Truncating mutations” included non-sense, splice site mutations, and frameshift insertion and deletion; “Non-truncating mutations” included missense mutations and inframe insertion and deletion.

The genomic mutational landscape of PBRM1 and clinical characteristics is displayed in Figure 5A. According to the TCGA pan-cancer cohort, PBRM1 mutation frequency was 5%, with the most prevalent in ccRCC (29.9%), followed by uterine corpus endometrial carcinoma (UCEC) (13.8%) and SKCM (9.3%) (Figure 5B). In addition, the most frequent mutation site found is I279Yfs*4 and I279Nfs*8 mutation (Figure 5C).

**FIGURE 6:**
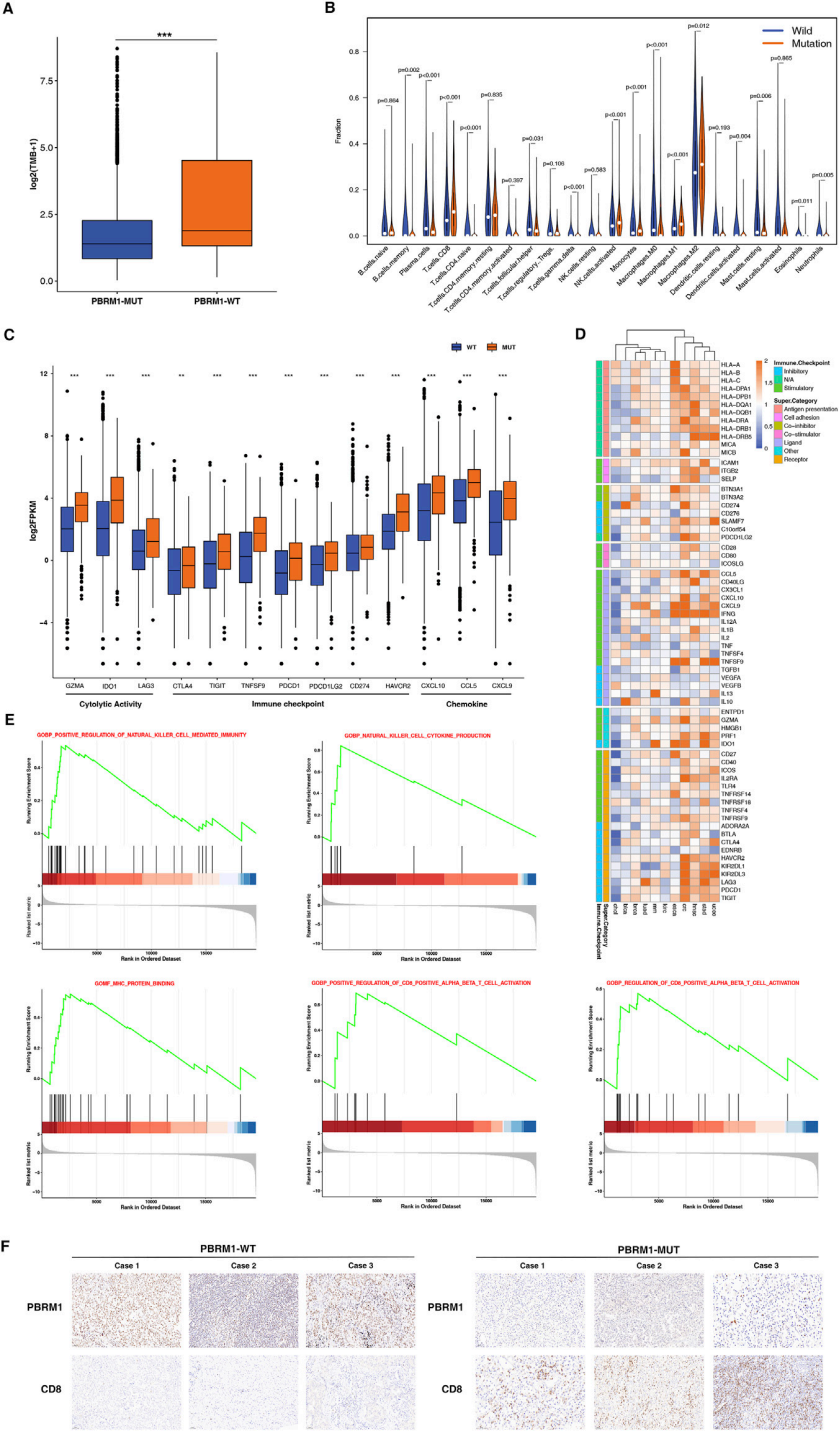
PBRM1-MUT was associated with enhanced anti-tumor immunity in TCGA cohort. **(A)** Comparison of the TMB between PBRM1-WT and PBRM1-MUT tumors in TCGA cohort. **(B)** Comparison of the immune cells infiltration levels in PBRM1-MUT and PBRM1-WT tumors. CIBERSORT was used to calculate the infiltration degree of these immune cells. **(C)** Boxplot depicting the expression level of immune-related genes in PBRM1-MUT and PBRM1-WT groups. **(D)** Heatmap shows change in the expression level of immune-related genes across multiple cancer types in PBRM1-MUT and PBRM1-WT. Blue indicated downregulation and orange indicated upregulation. **(E)** GSEA plot was used to analyse representative immune pathways identified by GSEA between PBRM1-MUTand PBRM1-WT tumors. **(F)** Quantitative immunohistochemistry (IHC) analysis of PBRM1 and CD8 protein expression in the PBRM1-MUT and PBRM1-WT groups (*n* = 3/group). **p < .01, ***p < .001, ****p < .0001.

To explore the potential mechanisms PBRM1-MUT in immunotherapy response, we first analyzed the relationship between PBRM1 and TMB. As shown in Figure 6A, the patients with PBRM1 mutations have higher TMB than the ones without PBRM1 mutations. CIBERSORT was used to investigate immune cell infiltration. The results found that CD8+T-cells, NK cells, monocytes, and M1 macrophages were enriched in the high mutational PBRM1-MUT group, in contrast CD4 naive T-cells, memory B cells were enriched in the PBRM1-WT group (Figure 6B). There was also an increase in the expression of genes related to cytotoxic activity (GZMA, IDO1, LAG3), checkpoint expression (CTLA4, TIGIT, TNFSF9, PDCD1, PDCD1LG2, CD274, HAVCR2), and chemokine expression (CXCL10, CCL5, CXCL9) (Figure 6C). To investigate the association between PBRM1 mutations and immune response in multiple cancers, we conducted compressive analyses of immune-related genes for each cancer type. The genes associated with defense and immune responses were significantly upregulated in PBRM1 mutation group. On the contrary, most of the genes involved in immunosuppression were significantly down-regulated (Figure 6D). Moreover, Gene set enrichment analysis (GSEA) was performed to identify potential immune-related pathways affected by PBRM1. The results were shown that the prominent enrichment of immune-related signal pathways to killer cell mediated immunity regulation, cell cytokine production, CD8^+^ T-cell activation in PBRM1-MUT group, compared with the PBRM1-MT groups (Figure 6E). Moreover, MHC protein binding related pathways were upregulated in PBRM1-MUT tumors (Figure 6E). As shown in Figure 6F, the expression of PBRM1 were decreased in PBRM1-MUT patients. Immunohistochemistry (IHC) staining of CD8 confirmed the presence of tumor-infiltrating T-cells in the PBRM1-MUT tumors. In addition, the expression of CD8 was markedly increased in PBRM1-MUT tumors compared to PBRM1-WT tumors. According to these findings, strongly associations between PBRM1 mutation and immune microenvironment may account for the good immunotherapeutic effect.

## Discussion

Cancer immunotherapy targeting immune checkpoint molecules has become a hot topic of research in recent years (Zhao et al., 2020). In spite of these prior researches have proved that the immunotherapeutic brings an obvious survival benefit (Ribas and Wolchok, 2018), the majority of patients fail to experience benefit (Hegde and Chen, 2020). Hence, it is still important to find novel biomarkers to predict the efficacy of immunotherapy in multiple cancers. Our previous study have found that KDR-MUT was associated with a better prognosis in pan-cancer patients who received ICIs (Cui et al., 2022). In the study, we found that PBRM1 mutation is strong correlation with prognosis and can serve as a predictor of immunotherapy.

PBRM1 encodes the BAF180 protein, which is important in chromatin remodeling (Lu and Allis, 2017; Zhou et al., 2020). PBRM1 genes had approximately 5% mutational frequency in cancer, while with high frequency of about 20%–40% in ccRCC(15). As noted by the previous study, PBRM1 mutation status could also be related to immunotherapy efficacy in the cancer, especially in ccRCC(15). The data from our study found that PBRM1 mutation frequency is about 5%, which was consistent with previous reports (Yang et al., 2021). A significant increase in the survival time were observed in the PBRM1-MUT group compared with the PBRM1-WT group. In addition, PBRM1 mutation can affect the immune microenvironment and the activation of immune-related pathways in cancer patients. Compared with the PBRM1-WT group, CD8 + T-cells, NK cells, monocytes, and M1 macrophages were enriched in the high mutational PBRM1-MUT group. Alternatively, PBRM1 mutation can activate a series of immune related pathway, including killer cell mediated immunity regulation, cell cytokine production, CD8^+^ T-cell activation. Perhaps for this reason, PBRM1 mutation is associated with a favorable outcome of immunotherapy in multiple cancers.

In our study, the relationship between PBRM1 and the prognosis of immunotherapy in pan-cancer was comprehensively analyzed by combining the universal carcinomatous public cohort and our clinical data collected in the First Affiliated Hospital of Nanjing Medical University. Nevertheless, there exist several limitations in our study. First of all, the mutation rate of PBRM1 is notable difference across the tumor subtypes. And the mutation rate of ccRCC is significantly higher than that in other tumors. That might be a critical confounding factor in this study. Secondly, we recognize that our sample size of NSCLC cohort is small, which affects the persuasiveness of the results. Therefore, we expect to obtain a larger sample size to confirm our findings. Third, all the immune cell infiltration and immune-related pathways was extracted from GSEA, hence specific molecular mechanisms have not been identified. In addition, we did not conducted a part of *in vitro* and *in vivo* experiments to verify the relationship between PBRM1 mutation and immunotherapy in cancer. Consequently, further in-depth research is needed to explore the mechanism by which PBRM1 mutation is associated with immune microenvironment.

In summary, the above were fully illustrated the links between the PBRM1 mutation and ICIs response in pan-cancer patients with treated ICIs. PBRM1 mutation be used to a promising immunotherapeutic signature that could guide clinical management and personalized immunotherapy.

## Data Availability

The original contributions presented in the study are included in the article/Supplementary Material, further inquiries can be directed to the corresponding authors.
