# Supplementary material for: PBRM1 mutation as a predictive biomarker for immunotherapy in multiple cancers
Source: Front Genet. 2023 Jan 9;13:1066347. doi: 10.3389/fgene.2022.1066347 (PMC9868445; doi:10.3389/fgene.2022.1066347)
Supplement: Supplementary file 1 [file DataSheet2.PDF]

Supplemental Information

Supplementary Table 1. Patient characteristics in the discovery cohort stratified by PBRM1 status

Characteristics	NO.(%)	PBRM1 status [No. (%)][a]	
		PBRM1-MUT	PBRM1-WT
**No. of patients**	571	48	523
**Median age, years (range)**	63 (18-90)	60 (29-79)	63 (18-90)
**Age**			
≥ 60	284(49.74)	22 (45.83)	262 (50.10)
< 60	205(35.90)	21 (43.75)	184 (35.18)
NA[b]	82(14.36)	5 (10.42)	77 (14.72)
**Sex**			
Male	352(61.65)	26 (54.17)	326 (62.33)
Female	219(38.35)	22 (45.83)	197 (37.67)
**Cancer type**			
BLCA	27(4.73)	2 (4.17)	25 (4.78)
NSCLC	147(25.74)	5 (10.42)	142 (27.15)
ccRCC	35(6.13)	19 (39.58)	16 (3.06)
SKCM	362(63.40)	22 (45.83)	340 (65.01)
**Drug class**			
PD-(L)1	156(27.32)	26 (54.17)	130 (24.86)
CTLA-4	316(55.34)	20 (41.67)	296 (56.60)
Combination	8(1.40)	0 (0)	8 (1.53)
NA[c]	91(15.94)	2 (4.17)	89 (17.01)
**Best overall response**			
CR	32(5.60)	4 (8.33)	28 (5.35)
PR	134(23.47)	19 (39.58)	115 (21.99)
SD	110(19.26)	12 (25.00)	98 (18.74)
PD	279(48.86)	13 (27.08)	266 (50.86)
NE[d]	16(2.80)	0 (0)	16 (3.06)
**Durable clinical benefit**			
DCB	202(35.38)	28 (58.33)	174 (33.27)
NDB	234(40.98)	10 (20.83)	224 (42.83)
NE[e]	135(23.64)	10 (20.83)	125 (23.90)
**TMB (cutoff 20%)**			
Low	458(80.21)	36 (0.75)	422 (80.69)
High	113(19.79)	12 (0.25)	101 (19.31)

Abbreviations: NA: not available, BLCA: bladder cancer, NSCLC: non-small cell lung cancer, ccRCC: clear cell renal cell carcinoma, SKCM: melanoma, CR: complete response, PR: partial response, SD: stable disease, PD: progressive disease, PD-(L)1: programmed cell death-1 or programmed deathligand, CTLA-4: cytotoxic T cell lymphocyte-4, NE: not evaluable, DCB: durable clinical benefit, NDB: no durable benefit. [a]Indicated percentage of PBRM1-MUT or PBRM1-WT patients in a given category (i.e. specific gender, specific age group).[b]Eighty-two patients with age were not reported. [c]Ninety-one patients with drug class were not reported. [d]Sixteen patients with best overall response not evaluable due to missing data. [e]One hundred thirty-five patients with durable clinical benefit not evaluable to missing data.
